# Effects of Manual Therapy and Mechanical Massage on Spinal Alignment, Extension Range of Motion, Back Extensor Electromyographic Activity, and Thoracic Extension Strength in Individuals with Thoracic Hyperkyphosis: A Randomized Controlled Trial

**DOI:** 10.1155/2020/6526935

**Published:** 2020-11-23

**Authors:** Sung-hoon Jung, Ui-jae Hwang, Sun-hee Ahn, Jun-hee Kim, Oh-yun Kwon

**Affiliations:** Department of Physical Therapy, Yonsei University, Wonju, Kangwon-Do, Republic of Korea

## Abstract

**Background:**

Manual therapy has been recommended to reduce and prevent musculoskeletal problems related to thoracic hyperkyphosis. With recent rapid technological developments, manual techniques can now be implemented by mechanical devices; hence, mechanical massage can manipulate the back muscles and mobilize the spine.

**Purpose:**

Here, we aimed to 1) determine the effects of mechanical massage and manual therapy and 2) compare their effects on spinal posture, extension range of motion, trunk extensor electromyographic activity, and thoracic extension strength in individuals with thoracic hyperkyphosis.

**Methods:**

Participants with thoracic hyperkyphosis were randomly assigned to the manual therapy (*n* = 16) or mechanical massage (*n* = 19) group. Each intervention was applied for 8 weeks. The participants' spinal posture, extension range of motion, trunk extensor electromyographic activity, and thoracic extension strength were measured before and after intervention.

**Results:**

Intergroup analyses revealed no significant differences in any variables. However, thoracic kyphosis angle, thoracic extension range of motion, longissimus thoracis electromyographic activity, iliocostalis lumborum pars lumborum activity, and thoracic extension strength differed significantly in intertime analyses. The results of paired *t*-test analysis showed that thoracic kyphosis angle, thoracic extension range of motion, longissimus thoracis electromyographic activity, and thoracic extension strength were significantly different after intervention in both groups (*p* < 0.05).

**Conclusions:**

Mechanical massage and manual therapy effectively improve thoracic kyphosis angle, thoracic extension range of motion, and thoracic extension strength. Therefore, mechanical massage is an alternative intervention to manual therapy for improving thoracic kyphosis angle, thoracic extension range of motion, and thoracic extension strength in participants with hyperkyphosis. This trail is registered with KCT0004527.

## 1. Introduction

Thoracic hyperkyphosis is defined as an excessive anteroposterior curvature of the thoracic spine (>40°) [1, 2]. One recent study reported that 38% individuals aged 20–50 years are at a high risk of developing thoracic hyperkyphosis considering their excessive exposure to digital media devices such as computers, tablets, and smartphones [3, 4]. Thoracic hyperkyphosis is often accompanied by functional limitations, decreased quality of life, increased mortality, and musculoskeletal alterations [5].

Considering that individuals with thoracic hyperkyphosis have a directional susceptibility to movement, they usually experience a long-term thoracic hyperkyphosis posture that results in various musculoskeletal changes [6, 7]. Thoracic hyperkyphosis possibly lengthens or stretches the erector spinae, resulting in decreased positional sense, decreased back extensor strength, and increased thoracic and lumbar extensor muscle activity imbalance [7–10]. Thoracic hyperkyphosis limits thoracic extension during trunk extension and promotes lumbar lordosis [7]. In individuals with limited thoracic extension, excessive lumbar extension during trunk extension may contribute to low back pain because it exerts abnormal compression and shear forces on the lumbar spine [11–13].

Previous studies have recommended manual therapy to reduce and prevent musculoskeletal problems related to thoracic hyperkyphosis [14, 15]. Manual techniques reduce pain, improve circulation, correct spinal alignment, facilitate joint movements, stretch shortened muscles, and release joint adhesions [16–18]. Kamali et al. (2016) reported that an 8-week program of manual therapy comprising massage, mobilization, muscle energy, and myofascial release decreased thoracic hyperkyphosis angle and increased muscle strength when applied to postural hyperkyphosis. Bautmans et al. (2010) confirmed that a 3-month program of spinal mobilization, taping, and exercise in elderly postmenopausal patients with thoracic hyperkyphosis decreased the thoracic kyphosis angle.

With rapid recent technological developments, manual techniques can be implemented using mechanical devices without the need for manual handling; hence, mechanical massage can be performed using a skilled manual technique at any time in a private, convenient, and controlled setting [19, 20]. Previous studies demonstrated the effects of mechanical massage on stress [21]; mental fatigue and cognitive function [22]; heart rate, blood pressure, and temperature [23]; muscle relaxation [19]; and muscle fatigue and sympathetic and parasympathetic balance [24]. Mechanical back massage releases the erector spinae while simultaneously gliding the thoracic spine vertically with a mobilization effect that extends by segment. Therefore, an improvement in spinal kinematics is one of the potential benefits of mechanical massage, but this is yet to be studied. Therefore, here, we aimed to 1) determine the effects of mechanical massage and manual therapy and 2) compare their effects on thoracic and lumbar spinal alignment, extension range of motion, trunk extensor electromyographic (EMG) activity, and thoracic extension strength in individuals with thoracic hyperkyphosis.

## 2. Materials and Methods

### 2.1. Participants

Potential participants with a slouched posture were examined and recruited according to the inclusion criteria. Ninety-four individuals with thoracic hyperkyphosis were recruited through Internet advertisements between July 2018 and August 2019. Participants with the following characteristics were included in the study: age 18–50 years and a thoracic kyphosis angle >40° [5] measured using a spinal mouse (Idiag AG, Fehraltorf, Switzerland). Individuals with the following characteristics were excluded: scoliosis and a history of spinal column fracture, spinal tumors and related malignancies, congenital spinal anomalies, cancer, or rheumatoid arthritis [14]. The ethics committee of Yonsei University approved the study, and all participants provided informed consent before inclusion. The study was ethically approved by the Yonsei University Wonju Institutional Review Board (1041849-201901-BM-019-01). Because the clinical research information service registration process is time consuming, in some cases, the university review board allows trial commencement based on the permission granted by the institution. The study was registered in the Clinical Research Information Service after the experiment (KCT0004527). All processes were performed in a laboratory setting in Seoul. Based on a pilot study, the required sample size was calculated by a priori power analysis using G*∗*Power 3.1 software (G*∗*Power Software Inc., Kiel, Germany). Using an effect size calculating the partial eta squared of thoracic kyphosis, the *α* level was set to 0.05. A pilot study with five participants in each group determined that a total sample size of 16 was required in each group to obtain a power of 0.95.

### 2.2. Randomization

**Figure 1:**
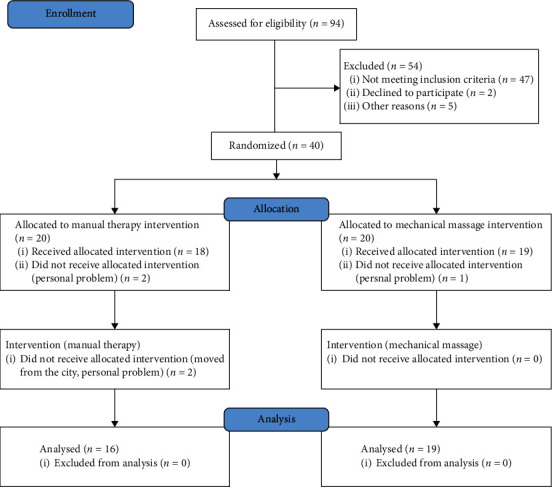
Flow diagram showing the participant enrollment, allocation, and analysis processes for the manual therapy and mechanical massage groups.

This randomized controlled trial investigated the effects of 8-week manual therapy and mechanical massage (24 sessions). Of the 94 participants, 40 participants met the inclusion criteria and were randomly assigned to the manual therapy group and mechanical massage group (*n* = 20 each). Microsoft Excel (Microsoft Corp., Redmond, WA, USA) was used to generate intervention assignments through a random number generated by reorganizing in a numerical ascending order. This process was performed by a principal investigator. A flow diagram showing participant enrollment, allocation, and analysis is shown in Figure 1. The characteristics of the participants are shown in Table 1.

### 2.3. Manual Therapy

The manual therapy protocol comprised massage, mobilization, and muscle energy, according to the protocol used by Kamali et al. (2016). Manual therapy reduces thoracic kyphosis and increases back extensor muscle strength [14]. This intervention begins with massage because the participants must be completely relaxed to ensure treatment efficacy [25, 26]. For the massage, the participant assumed the prone position. The physiotherapist applied wringing, skin rolling massage, and myofascial release to the back extensor muscles for 15 min [14]. For the mobilization, the patient assumed the prone position on a treatment table. The therapist placed his/her fist in the midsection of each spinous process of the thoracic vertebrae and applied downward pressure toward thoracic extension [14]. In each session, grade IV mobilization was performed with 40 repetitions on the middle thoracic spine (T4–T9) and 10 repetitions on the remaining thoracic vertebrae (T1–3, 10–12). For the muscle energy technique, the patient was instructed to sit in a relaxed position with both hands on the neck. The upper arms were supported on the therapist's arm. The therapist's other hand was placed on the spinous process of T7 to move the vertebra to the end of the extension barrier. Subsequently, the participant was instructed to maintain thoracic extension (not greater than approximately 25% of the maximum contraction) [17]. The therapist resisted the isometric contraction for 5 s. After 5 s isometric contraction, the participant was instructed to complete the relaxation. When the participant ceased the contraction, the therapist moved the thoracic vertebra into further extension. This technique was performed five times per session [17]. Manual therapy was applied in a university laboratory setting by the same physiotherapist in all sessions.

### 2.4. Mechanical Massage

**Figure 2:**
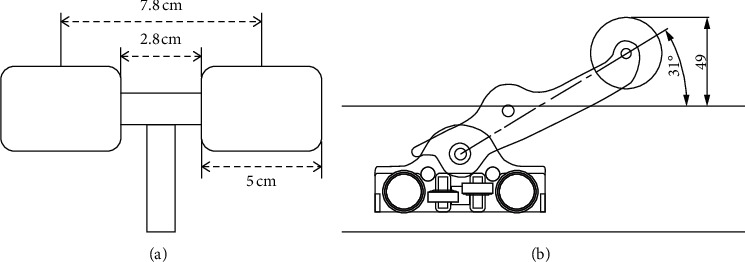
A mimetic diagram of the mechanical massager used in the thoracic region. (a) Superior view; (b) lateral view.

A Pookjam mechanical massage device (Factorial Inc., Seoul, Korea) was used. This mat-type mechanical massage device has two circular rollers measuring 5 cm in diameter located 7.8 cm between the midpoints of each roller. The distance between the medial end points of each roller was 2.8 cm (Figure 2(a)). This roller massages the bilateral erector spinae groups and simultaneously mobilizes the spine in the extension direction. The thoracic mode embedded in the device was applied to the thoracic spine with a rolling massage. This thoracic mode provides a massage from the head to the thoracic spine. The rollers drive up and down at 0.4 cm/s. In the thoracic mode, a total of three round drives are performed for 25 min. The roller moves up 4.9 cm from the mat in the thoracic area (Figure 2(b)).

### 2.5. Outcomes

#### 2.5.1. Radiographs

Digital lateral thoracic spine radiographs were obtained using a collimator (Medien International, Co., Ltd., Gyeonggi-do, Korea) and MC-D computed radiographic system (Medien International, Co., Ltd.). To obtain the radiographs, each participant stood with their left side adjacent to a radiographic bucky (with a 35 × 43-cm portrait-orientated computed radiography cassette enclosed). The X-ray beam was centered at T7 for the thoracic spine, including T12 superiorly and S3 inferiorly for the lumbar spine. A standard focal film, with a 100 cm distance, was used to collect all radiographs [27, 28]. Collimation was restricted to the skin edges posteriorly and was less than 13–15 cm in diameter (depending on participant size). Each participant was instructed to remain still while the X-ray image was generated during a suspended respiration. One lateral spine radiograph was obtained in the neutral standing posture, as well as one in the full spine extension posture. For lateral spine radiographs, each participant was instructed to stand in a natural (habitual) posture with the arm elevated to 70° [29]. For the extension movements, the participant wrapped both hands behind the head with the upper arms against the ears. Moreover, the participants were instructed to “point the elbows toward the ceiling and arch backward.” To promote spinal movement, they were asked to focus their movement on the midthoracic region during each movement [27, 30].

**Figure 3:**
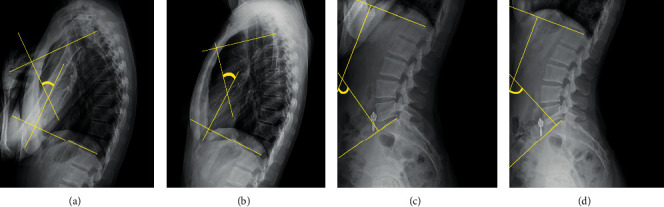
Lateral radiographs showing thoracic kyphosis (a), thoracic extension (b), lumbar lordosis (c), and lumbar extension (d). The line markers indicate the Cobb angle method used to take measurements in each position.

All image analyses were performed by one investigator who also performed a repeatability study. Analysis of the radiographic images was performed on a personal computer using ImageJ software (National Institutes of Health, Bethesda, MD, USA). Thoracic kyphosis and lumbar lordosis were measured with the participants in the neutral position, and the thoracic and lumbar extension angles were measured with the participants in the fully extended position using the Cobb angle technique [27, 30]. For thoracic kyphosis and extension, lines were extended from the superior end plate of the T4 vertebral body and from the inferior end plate of the T12 vertebral body [31]. If the superior end plate of T4 was not confirmed due to the humeral head position, the inferior end plate of T4 was analyzed. For lumbar lordosis and extension, the lines were extended from the superior end plate of the first lumbar vertebra and the superior line of the sacrum [32]. The angle formed by the intersection of the lines drawn perpendicular to these lines was used to define the Cobb angle (Figure 3).

#### 2.5.2. Surface Electromyography

Erector spinae muscle activity was measured according to a previous study on individuals with thoracic hyperkyphosis [7]. Iliocostalis lumborum pars lumborum, longissimus thoracis, and iliocostalis lumborum pars thoracis activations were measured using a Tele-Myo Direct Transmission System electromyograph fitted with a wireless telemetry system (Noraxon Inc., Scottsdale, AZ, USA) according to Criswell [33]. The iliocostalis lumborum pars lumborum muscle was attached at the L3 level midway between the lateralmost palpable border of the erector spinae and a vertical line through the posterosuperior iliac spine. The attachment position of the longissimus thoracis was the T9 level, midway between a line through the spinous process, and a vertical line through the posterosuperior iliac spine, located approximately 5 cm laterally. Furthermore, the iliocostalis lumborum pars thoracis muscle was attached at the T10 level midway between the lateralmost palpable border of the erector spinae and a vertical line through the posterosuperior iliac spine. Skin impedance was reduced via the shaving of excess body hair if necessary, gently abrading the skin with fine grade sandpaper, and wiping the skin with alcohol swabs.

Signals were collected using MyoResearch® XP Master Edition software (Noraxon Inc.). The raw EMG signals were band-pass filtered (20–450 Hz), and the root mean square values were calculated using a 50 ms window. The data were recorded at a sampling rate of 2048 Hz.

**Figure 4:**
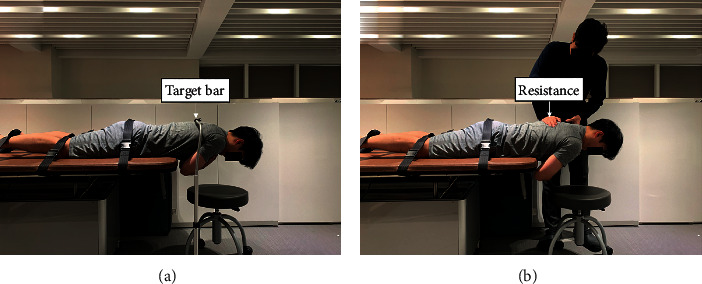
Measurement of surface electromyography activity during back extension. (a) Back extension; (b) maximum voluntary isometric contraction.

EMG activity was collected when participants maintained their back extension in the prone position. Back extension in the prone position was performed when the xiphoid process was aligned at the table edge, the participants raised their hands across their chest, and the lower extremities (hip, knee, and ankle) were fixed with nonelastic straps. While looking down, the participants were instructed to raise their bodies to the target bar horizontally (parallel to the ground) and hold this position for 5 s (Figure 4(a)). This target bar was set to the T6 level when the participants lay down on the table. This process included a 3 min rest between the two sessions. EMG activity was expressed as % maximum voluntary isometric contraction (MVIC), and the MVIC measurements were performed as described previously (Figure 4(a)) [7]. To measure the MVIC, back extension was performed with maximum isometric effort against the examiner's resistance (Figure 4(b)).

#### 2.5.3. Thoracic Extension Strength

**Figure 5:**
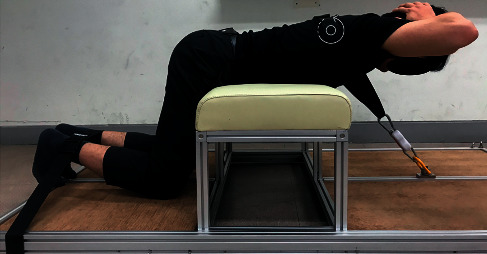
Measurement of thoracic extension strength.

Thoracic extensor strength was measured using a Smart KEMA tension sensor (KOREATECH Co., Ltd, Seoul, Korea). The tension sensor had a measurement range of 0–1, 960 N, with an accuracy of 4.9 N and a sampling rate of 10 Hz. To measure thoracic extensor strength, participants flexed the knee to 90° in the prone position using a custom-built apparatus [14]. One end of the orthopedic belt was connected to a fixed vertical bar in front of the participant's chest at the T6–T7 level, while the other end was connected to a chest harness. The participants were asked to perform the maximum possible thoracic extension (Figure 5) [34]. Each participant performed two maximum 5-s isometric contractions with a 30 s rest between them. The examiner, who was blinded to the treatment group allocations, recorded the mean value between 2 and 4 s.

### 2.6. Statistical Analysis

The Kolmogorov–Smirnov test was used to assess the homogeneity of variance of radiography, EMG activity, and strength (*p* > 0.05). Moreover, an independent *t*-test was used to evaluate the intergroup differences in initial values of all variables. If no difference was noted in the initial values, a 2 × 2 repeated measure analysis of variance was performed of the factors in the intervention group (manual therapy and mechanical massage) and time (before and after intervention) to compare the effects between groups. If a significant group × time interaction was identified, post hoc analysis was performed. In the analysis of variance, partial eta squared was used as the effect size [35]. To determine the effects of the intervention by group, the paired *t*-test was performed. In the paired-t test analysis, Cohen's d was used as the effect size and calculated as the difference between the means divided by the pooled standard deviation [36]. All statistical analyses were performed using the statistical software package Statistical Package for the Social Sciences (SPSS) version 18.0 (SPSS Inc., Chicago, IL, USA), and the level of statistical significance was set at *p* < 0.05.

## 3. Results

Forty participants with thoracic hyperkyphosis were randomly divided into the manual therapy and mechanical massage groups (*n* = 20 each); however, for personal reasons, five participants withdrew before or during intervention (four in the manual therapy group and one in the mechanical massage group). Ultimately, 16 participants in the manual therapy group and 19 participants in the mechanical massage group completed the intervention and were included in the analysis (Table 1). There were no significant intergroup differences in sex, age, height, mass, body mass index (Table 1), or preintervention values of any variables (*p* > 0.05). Two-way analysis of variance revealed no significant group-by-intervention interactions for all variables and revealed significant group-by-intervention interactions for thoracic kyphosis angle (*F* = 27.657, *p* ≤ 0.001), thoracic extension range of motion (*F* = 41.361, *p* ≤ 0.001), longissimus thoracis EMG activity (*F* = 10.912, *p* = 0.002), iliocostalis lumborum pars lumborum EMG activity (*F* = 4.700, *p* = 0.037), or thoracic extension strength (*F* = 105.362, *p* ≤ 0.001) (Table 2). The results of paired *t*-test analysis showed that the thoracic kyphosis angle, thoracic extension range of motion, longissimus thoracis activity, and thoracic extension strength were significantly different before intervention in both groups (*p* < 0.05) (Table 3).

## 4. Discussion

Various manual therapies have been considered effective at reducing thoracic hyperkyphosis. However, these therapies require a sufficient amount of therapist and patient time. If these therapies can be performed using a mechanical device, they can be applied conveniently, saving both therapist and patient time. In this study, the effects of manual therapy versus mechanical massage were identified and compared. Both interventions were effective in improving the thoracic kyphosis angle, thoracic extension range of motion, and thoracic extension strength. Furthermore, no intergroup difference was detected.

Posteroanterior mobilization provided by manual therapy extends the thoracic segments [37]. Thoracic hyperkyphosis involves excessive thoracic flexion, with the facet joints gliding forward and upward [38]. Therefore, posteroanterior mobilization reduces the kyphosis angle by restoring normal segment mobility [37]. Previous studies reported a 3.4° improvement (from 52.5° to 49.1°) when 18 sessions of spinal mobilization, taping, and exercise were applied [39]. Kamali et al. (2016) reported a 5.1° improvement (from 45.7° to 40.6°) when an 8-week program of manual therapy consisting of massage, mobilization, and muscle energy and myofascial release techniques was applied. In this study, the manual therapy group showed a 5.31° improvement after 8 weeks of intervention, consistent with the results of a previous study. The mechanical massage group also showed significant (4.32°) improvement. Furthermore, there was no significant intergroup difference in the effects. Based on these results, mechanical massage is also considered effective at reducing thoracic kyphosis, making it a valid alternative intervention.

Extension mobility of the thoracic spine is important for optimizing the patterns of load transfer and facilitating the functional movement of the thoracolumbar spine and shoulder girdle [40, 41]. The thoracic extension range of motion improved in both groups after an 8-week intervention, possibly due to restoration of normal segment mobility [37]. However, no significant difference was noted in lumbar lordosis or active extension angle after an 8-week intervention in this study. In previous studies, thoracic intervention improved cervical spine alignment and range of motion [42, 43]. However, to our knowledge, no study has reported that thoracic intervention affects lumbar spine alignment and range of motion. This may be because alterations of the lower segment may affect the upper segment more than the opposite in the upright spinal position.

Manual therapy can improve paraspinal activity and thoracic extension strength [14]. In this study, the maximum isometric strength of thoracic extension improved in both groups after intervention. Mobilization of the restricted thoracic segment joint would have regained its strength as the segment mobility of the thoracic extension recovered [14]. The %MVIC of the longissimus thoracis muscle decreased significantly during thoracic extension after intervention. As the extension mobility of the thoracic spine improved, less effort was required of the longissimus thoracis when the thorax moved up to the target bar during back extension. Our results support the findings reported Pecos-Martin et al. (2017), who reported that mobilization reduced thoracic erector spinae activity during back extension. [44]

Individuals with thoracic hyperkyphosis have increased lumbar lordosis during back extension compared to those without thoracic hyperkyphosis [7]. This suggests that individuals with thoracic hyperkyphosis predominantly activate their lumbar extensors during back extension. In our study, the iliocostalis lumborum pars lumborum EMG activity during back extension was significantly decreased after an 8-week intervention, while the iliocostalis lumborum pars thoracic EMG activity did not differ significantly. These results suggest that the two interventions reduced the dominant activity of the lumbar extensor during back extension in individuals with thoracic hyperkyphosis. Our results also support the findings reported by Park et al. (2015); they reported that individuals with thoracic hyperkyphosis showed greater muscle activity imbalances between the iliocostalis lumborum pars lumborum and the longissimus thoracis muscles during back extension compared to those without thoracic hyperkyphosis.

Our study has several limitations. First, no follow-up information was obtained. Hence, we could not determine the long-term effects of manual therapy and mechanical massage. Thus, further studies are required to determine the long-term effects of these therapies. Second, the intervention was applied to individuals with thoracic hyperkyphosis (40.88° in the manual therapy group, 43.43° in the mechanical massage group). Therefore, the study findings cannot be generalized to individuals with more severe thoracic hyperkyphosis. Third, we did not enroll older participants (aged >50 years). Thus, our findings cannot be generalized to elderly populations.

## 5. Conclusions

The thoracic kyphosis angle, thoracic extension range of motion, and thoracic extension strength improved in individuals with thoracic hyperkyphosis after an 8-week intervention of manual therapy or mechanical massage. Moreover, both interventions effectively improved the thoracic kyphosis angle, thoracic extension range of motion, and thoracic extension strength. Therefore, mechanical massage is an alternative intervention to manual therapy for improving the thoracic kyphosis angle, thoracic extension range of motion, and thoracic extension strength of participants with hyperkyphosis.

## Figures and Tables

**Table 1:** Demographic data.

	Manual therapy, mean ± SD	Mechanical massage, mean ± SD	Statistics	*p*
Age (y)	35.94 ± 7.18	33.37 ± 6.56	*t* = –1.106	0.277
Sex, male/female	8/8	12/7	*χ* ^2^ = –0.614	0.433
Mass (kg)	67.20 ± 17.23	69.77 ± 14.70	*t* = 0.476	0.637
Height (cm)	167.68 ± 7.21	170.32 ± 7.92	*t* = 1.023	0.314
BMI (kg/m^2^)	23.77 ± 4.63	23.83 ± 3.66	*t* = 0.045	0.964
Thoracic kyphosis^(º)^	40.88 ± 5.65	43.43 ± 6.76	*t* = 1.197	0.240
Lumbar lordosis^(º)^	55.65 ± 9.88	53.99 ± 9.00	*t* = –0.520	0.607

BMI, body mass index; SD, standard deviation.

**Table 2:** Mixed model analysis of variance results for the outcome measures.

		Group				Time				Group × time		
		*F*	*p*	Effect size	95% CI	*F*	*p*	Effect size	95% CI	*F*	*p*
Spinal posture	Thoracic kyphosis^(º)^	2.181	0.149	0.062	−1.152 to 7.251	27.657	≤0.001^*∗*^	0.456	2.949 to 6.671	0.296	0.590
	Lumbar lordosis^(º)^	0.019	0.890	0.001	−6.244 to 5.443	0.714	0.404	0.021	−1.182 to 2.860	1.608	0.214

Extension range of motion	Thoracic extension^(º)^	3.207	0.082	0.089	−0.708 to 11.113	41.361	≤0.001^*∗*^	0.556	5.254 to 10.116	3.834	0.059
	Lumbar extension^(º)^	3.584	0.067	0.098	−0.472 to 13.115	1.447	0.238	0.042	−1.354 to 5.274	2.569	0.118

Muscle activity	Longissimus thoracis (%MVIC)	0.666	0.420	0.020	−0.084 to 0.036	10.912	0.002^*∗*^	0.248	0.021 to 0.086	0.105	0.747
	Iliocostalis lumborum pars thoracis (%MVIC)	0.730	0.399	0.022	−0.083 to 0.034	0.547	0.465	0.016	−0.021 to 0.045	0.662	0.422
	Iliocostalis lumborum pars lumborum (%MVIC)	0.356	0.555	0.011	−0.056 to 0.102	4.700	0.037^*∗*^	0.125	0.002 to 0.072	0.227	0.637

Strength (kgf)		1.025	0.319	0.030	−6.355 to 18.943	105.362	≤0.001^*∗*^	0.761	−22.556 to −15.093	0.728	0.400

CI, confidence interval; MVIC, maximum voluntary isometric contraction. ^*∗*^*p* < 0.05.

**Table 3:** Outcome measure changes before intervention versus after intervention by group.

		Manual therapy				Mechanical massage			
		Before intervention, mean ± SD	After intervention, mean ± SD	*p*	Effect size	Before intervention, mean ± SD	After intervention, mean ± SD	*p*	Effect size
Spinal posture	Thoracic kyphosis^(º)^	40.88 ± 5.65	35.57 ± 7.32	0.001^*∗*^	0.812	43.43 ± 6.76	39.11 ± 6.74	0.003^*∗*^	0.640
	Lumbar lordosis^(º)^	55.65 ± 9.88	53.55 ± 8.44	0.151	0.229	53.99 ± 9.00	54.41 ± 8.51	0.767	0.048

Extension range of motion	Thoracic extension^(º)^	31.91 ± 8.95	21.88 ± 12.40	≤0.001^*∗*^	0.928	34.77 ± 7.61	29.42 ± 7.84	0.002^*∗*^	0.692
	Lumbar extension^(º)^	52.93 ± 10.55	48.36 ± 13.86	0.195	0.371	56.64 ± 9.91	57.30 ± 9.41	0.510	0.068

Muscle activity	Longissimus thoracis (%MVIC)	0.31 ± 0.10	0.26 ± 0.08	0.046^*∗*^	0.552	0.29 ± 0.13	0.23 ± 0.07	0.020^*∗*^	0.575
	Iliocostalis lumborum pars thoracis (%MVIC)	0.37 ± 0.10	0.37 ± 0.10	0.962	0	0.36 ± 0.11	0.33 ± 0.08	0.255	0.312
	Iliocostalis lumborum pars lumborum (%MVIC)	0.50 ± 0.12	0.47 ± 0.93	0.313	0.045	0.53 ± 0.14	0.48 ± 0.14	0.045^*∗*^	0.357

Strength (kgf)		30.18 ± 12.73	47.44 ± 15.49	≤0.001^*∗*^	1.217	34.91 ± 20.13	55.30 ± 24.45	≤0.001^*∗*^	0.910

MVIC, maximum voluntary isometric contraction; SD, standard deviation. ^*∗*^*p* < 0.05.

## Data Availability

Data are available on request from the corresponding author.
